# [Retracted] Allicin suppresses the migration and invasion in cervical cancer cells mainly by inhibiting NRF2

**DOI:** 10.3892/etm.2024.12659

**Published:** 2024-07-23

**Authors:** Qiumei Zhang, Dongmei Yang

Exp Ther Med 17:1523–1528, 2019; DOI: 10.3892/etm.2018.7104

Following the publication of the above paper, it was drawn to the Editor’s attention by a concerned reader that certain of the DHE-stained images shown in Fig. 2A, the cell migration and invasion assay data in Fig. 4A and B, the flow cytometric assay data in Fig. 4C, and certain of the western blotting data shown in Fig. 5A and the cell apoptotic data in Fig. 5B were strikingly similar to data that had already appeared in previously published articles written by different authors at different research institutes. Owing to the fact that the contentious data in the above article had already been published prior to its submission to *Experimental and Therapeutic Medicine,* the Editor has decided that this paper should be retracted from the Journal. The authors were asked for an explanation to account for these concerns, but the Editorial Office did not receive a reply. The Editor apologizes to the readership for any inconvenience caused.

